# A New Mode of Taxis for Strangulated Hernia

**Published:** 1875-03

**Authors:** Augustus F. Erich

**Affiliations:** Professor of Diseases of Women and Children, College of Physicians and Surgeons, Baltimore, Maryland


### A NEW MODE OF TAXIS FOR STRANGULATED HERNIA.

BY AUGUSTUS F. ERICH, M.D.,

*Professor of Diseases of Women and Children, College of Physicians and Surgeons, Baltimore, Maryland.*

The following history of a case of strangulated hernia successfully treated by what I call the postural taxis, is published for the purpose of enlisting the aid of the profession generally in determining, by more numerous experiments than one person would have the opportunity of making, the true value of what seems to be a valuable discovery.

The posture assumed in this case would so naturally suggest itself to any person's mind, that it is probable that it has been tried before. But as I have never seen it used or suggested, and as five treatises on surgery—including Gross's and Erichsen's—which I have consulted, are silent on the subject, I feel it my duty to communicate it.

Mr. K., æt. sixty-three years, watchman at a steamship wharf, had been suffering from a right inguinal hernia about two years, for which he wore no truss. He was suddenly seized with violent symptoms of strangulation at 4 A.M., December 18th, 1874, while at his post. Finding himself unable to procure assistance, he was obliged to lie down on the wharf, where he was found three hours later in a state of semi-unconsciousness produced by pain and cold. He was removed to his home, and my friend Dr. R. W. Mansfield called in to reduce the hernia. He administered chloroform and practiced the taxis, assisted by two neighbors, until he was completely exhausted. I was then called in consultation, and after the reädministration of chloroform to a complete state of relaxation, the taxis in the usual method was practiced by us, assisted by two men, as long as we deemed it safe. All our efforts having failed, we prescribed an opiate, and the patient being still very cold, ordered warm fomentations to the hernia, and agreed to meet again at 2 P.M. of the same day. Having met according to appointment, chloroform was once more administered, and the taxis tried with the same assistance as before, without producing the slightest change in the hernial tumor. The patient having vomited several times, there seemed to be no other alternative than an operation. Knowing, however, the fearful death-rate after that operation, especially in persons of such advanced age (sixty-three years), I cast about for other expedients that might avert our patient's impending doom.

Knowing that contraction as well as tumefaction in any part of the body can most readily be overcome by a gentle but continuous force, if applied for a sufficient length of time, while it would resist a much greater force applied intermittingly, it occurred to me that if we could put the patient's body in such a position as to cause the intestines to gravitate toward the chest, we would apply just such a gentle continuous force as would be most likely to cause relaxation of the inguinal ring. Acting upon this theory, a couple of short boards were laid against the side of the bed, and secured in such a position as to form an inclined plane at an angle of 45 degrees with the floor. These boards were then covered by a mattress, and the patient placed upon it, with his legs resting upon the bed, and his head upon a pillow on the floor. Reaction being now sufficiently established, we ordered an ice-bladder to the ring, and arranged for another meeting a few hours later for the purpose of operating. The patient had been in this position a little over an hour, when we received the joyous tidings that the tumor had disappeared, and upon an examination the hernia was found to be completely reduced by the force of gravity.

Although the ice applied to the inguinal ring may have assisted in diminishing tumefaction of the parts, I have used it too frequently, without success in former cases, to attribute much of the result to its influence.

The *modus operandi* of this postural taxis seems to be as follows:

1st. In placing the patient in this position we remove all pressure against the inguinal opening from the interior of the abdomen.

2d. We make gentle traction upon both ends of the protruding intestine from the inside of the abdomen.

3d. The whole weight of the hernial tumor is brought to bear upon the inguinal ring.

4th. The natural elasticity of all the coverings of the hernia will have a tendency to assist in forcing the contents of the sack back into the abdominal cavity.

Since the recovery of this patient, an additional expedient has suggested itself to my mind, which I intend to try on the next case of strangulated hernia that may present itself. It consists in placing a thin bag of fine sand of a suitable shape upon the tumor, so as to apply as much pressure exteriorly as the patient may be able to bear.

I conclude with the most earnest request to all my professional brethren to give this mode of taxis a trial, and report their failures as well as successes, so that we may soon ascertain the true value of this operation.

---

*Lemon Juice in Diphtheria.*—M. Revillout (*Gaz. des Hôpitaux*) recommends, in the strongest terms, the employment of large quantities of pure lemon juice as a gargle. He says that he and his father have used it during eighteen years, and always with success, it being the most certain application yet known.—*Medical Times and Gazette.*

